# The Surge of Metal–Organic-Framework (MOFs)-Based Electrodes as Key Elements in Electrochemically Driven Processes for the Environment

**DOI:** 10.3390/molecules26185713

**Published:** 2021-09-21

**Authors:** Abdoulaye Thiam, Juan A. Lopez-Ruiz, Dushyant Barpaga, Sergi Garcia-Segura

**Affiliations:** 1 Programa Institucional de Fomento a la Investigación, Desarrollo e Innovación (PIDi), Universidad Tecnológica Metropolitana, Ignacio Valdivieso 2409, San Joaquín, Santiago 8940577, Chile; athiam@utem.cl; 2 Institute for Integrated Catalysis, Pacific Northwest National Laboratory, 902 Battelle Blvd, Richland, WA 99352, USA; juan.lopezruiz@pnnl.gov; 3 Energy and Environment Directorate, Pacific Northwest National Laboratory, 902 Battelle Blvd, Richland, WA 99352, USA; dushyant.barpaga@pnnl.gov; 4 Nanosystems Engineering Research Center for Nanotechnology-Enabled Water Treatment, School of Sustainable Engineering and the Built Environment, Arizona State University, Tempe, AZ 85287, USA

**Keywords:** water treatment, electrochemical advanced oxidation processes (EAOPs), photoelectrocatalysis, electro-Fenton, capacitive deionization (CDI), electrocatalysis, metal–organic framework (MOF), carbonized metal–organic framework (MOFC), zeolitic imidazolate frameworks (ZIF)

## Abstract

Metal–organic-frameworks (MOFs) are emerging materials used in the environmental electrochemistry community for Faradaic and non-Faradaic water remediation technologies. It has been concluded that MOF-based materials show improvement in performance compared to traditional (non-)faradaic materials. In particular, this review outlines MOF synthesis and their application in the fields of electron- and photoelectron-Fenton degradation reactions, photoelectrocatalytic degradations, and capacitive deionization physical separations. This work overviews the main electrode materials used for the different environmental remediation processes, discusses the main performance enhancements achieved via the utilization of MOFs compared to traditional materials, and provides perspective and insights for the further development of the utilization of MOF-derived materials in electrified water treatment.

## 1. Introduction

Access to clean water is identified by the United Nations as one of the major sustainable challenges of this century [1]. Anthropogenic activity has accelerated the deterioration of natural and built environment, resulting in limited access to water sources due to their pollution. The availability of drinking water is aggravated by climate change in water stressed communities worldwide. Technologies that enable water reuse are emerging as the most feasible solution to maximize usage of water in different contexts. 

Centralized water treatment has proven useful over the decades, but several shortcomings and the increasing widespread of pollutants of anthropogenic origin are challenging traditional water treatment systems. Recent challenges have been faced regarding not only the water treatment itself, but the water quality control during water delivery due to failure of conventional pipelines. The development of versatile technologies that are compact and user-friendly are an urgent need. The decentralization of water purification is a promising approach that can ensure access to high quality water by final users connected to the grid and those relying on alternative water supplies such as wells. The electrification of water technologies can provide a holistic solution to the challenges of access to clean water and sanitation worldwide.

Similar challenges are being faced at industry level. Technologies that can ensure minimization of water usage in closed loops while aiming towards transition to zero liquid discharge practices are required. Treatment technologies with small physical footprints that can include different modular systems to deal with different target pollutants could be a promising alternative to large biological treatment systems. 

Electrochemical technologies are emerging as a promising solution given their versatility and efficient removal of organic pollutants, oxyanions, scalants, and even pathogens from water. However, challenges related to effective and selective electrode materials still exist [2]. In this context, metal–organic-frameworks (MOFs) are defining a new scientific pathway to overcome such barriers. This review introduces MOFs to the environmental electrochemistry community by describing the most fundamental concepts regarding their synthesis and structure. The description of unique properties of highly organized MOFs are used to connect with electrochemical applications based on electrocatalysis, photoelectrocatalysis, and non-faradaic processes. Advances on these different remediation technologies by the use of MOFs are presented from a critical perspective that also identifies urgent research questions to enhance MOF-based electrodes. 

## 2. Metal–Organic Frameworks: Synthesis, Structure, and Properties

**Figure 1:**
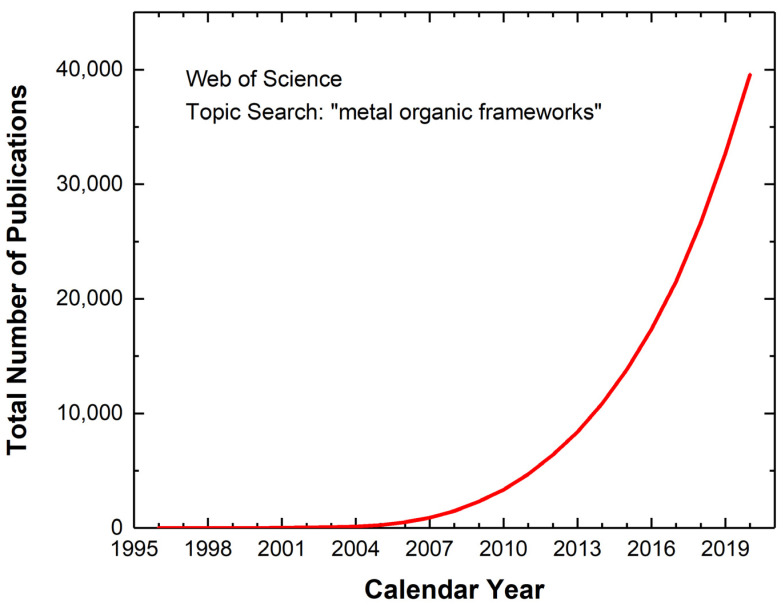
Total number of publications in scientific literature over the past 20+ years for “metal organic frameworks” as per Web of Science topic search engine.

Porous synthetic materials are increasingly being utilized for targeted applications involving separations, filtration, catalysis, sensing, storage, etc. Such a wide range of use afforded by the molecular level systematic control of their chemistries has allowed researchers to probe and optimize them for desired textural, thermophysical, mechanical, chemical, and even electrical properties. These technological advancements continue to yield numerous evaluations of porous synthetic materials for their potential to revolutionize many industries including but not limited to chemicals, electronics, medical, biological, energy, and environmental. Among the most versatile from this faction of reticular material chemistry are a relatively new class of porous synthetic materials called MOFs [3,4,5]. As implied by their name, MOFs are composed of two fundamental components, metal nodes and organic linkers, that self-crystallize to form a highly ordered network [6]. Their relative simplicity of synthesis in tandem with the abundance of node-linker combinations has yielded thousands of unique MOFs for a multitude of applications, as depicted in Figure 1, by the rapid increase in recent literature for this field. As expected, precise control over the properties of these base ingredients can translate to the resulting crystallized framework and is the general methodology for designing MOFs. This unprecedented molecular level tailorability represents the fundamental advantage over other porous materials that are limited by their chemical composition (such as zeolites, carbons, aluminas, silicas, etc.) [7]. Overall, this large degree of freedom in synthetic control to produce a highly porous solid network with virtually any imaginable chemistry allows researchers to continually expand the application of MOFs [8].

**Figure 2:**
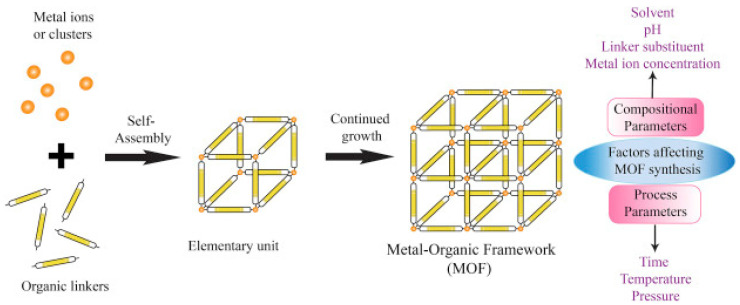
Total schematic of the building blocks of MOFs where metal clusters/SBUs (circles) coordinate with organic linkers (pegs/sticks) to form a homogenous repetitive crystalline framework [9].

Typically, MOF synthesis is performed in a one-pot batch reaction using some combination of solvent, stirring, and/or heat to promote the kinetics of self-crystallization between the metal salt and the organic monomer (see Figure 2). As the crystals develop and agglomerate into granules/powders, they crash out of solution and are easily separated. The solvent used in synthesis typically remains within the pores of the as-collected crystalline framework. Upon solvent exchange with a more volatile alternative followed by vacuum assisted desorption, these pores can be “cleaned” out to leave behind an immense void pore volume. This “activated” MOF material is then utilized to target the adsorption of desired analytes or as a base for synthesis of highly organized carbon structures through calcination. It should be noted that some non-traditional protocols, including mechanochemical synthesis (lack of solvent) and continuous flow-based synthesis (not limited to batch size), have also gained attention in literature given their favorable scalability considerations.

The metal constituents of the MOF usually comprise the transition and post-transition metal series of elements that are introduced as salts with positive oxidation states that coordinate to the organic linkers via metal oxide bridges. These bridges form a secondary building unit (SBU), typically of multiple metal oxide, to form the node/cluster [10]. Unlike traditional inorganic porous solids, such as zeolites, where the SBU coordinates with an anionic species (SiO_4_, PO_4_, SO_4_, etc.), in MOFs, the anionic species is replaced by an organic linker [6]. It should be noted that MOF nodes with unique elements from across the periodic table have also been synthesized [11,12,13]. This variation in nodes typically impacts analyte affinity to induce electrostatic interactions as well as the thermal and chemical stability of the MOF. The organic linkers vary greatly in size and composition but are usually comprised of at least two coordination sites to bind with SBUs to form a repetitive polymer-like network [14]. Examples of ligands include anionic species, such as carboxylic acid-based linkers (fumaric acids, various sized benzene-containing acids, large porphyrin compounds, etc.) that deprotonate in solvent to coordinate with SBUs, or neutral species, such as pyridine or nitrile-based linkers [14]. This variation in organic linkers has obvious impacts in pore size but can also impart unique chemical affinity via linker-coordinated moieties anchored in the pore to induce overall MOF functionality and stability. The synthesized structure of the MOF is typically experimentally characterized by its crystallinity (via X-ray diffraction), its porosity (via low temperature gas sorption), and its chemical environment (elemental composition, infrared, and/or nuclear magnetic resonance spectra) [10]. 

Since these monomers that make up the linkages of the MOF framework are on the order of nanometers in size, the resulting framework is also nanoporous. Numerous morphologies of these ordered pores have been shown, ranging from 2D channels to 3D cages with various smaller window-like pores into larger voids [15]. These highly homogenous networks of pores yield unique topologies but, in general, all have extremely high surface areas and total pore volumes [16]. Uniquely, the crystallinity and ordered nature of the frameworks result in porous solids without any non-accessible dead volumes [7]. These characteristics alone provide advantages over amorphous porous materials and have implications such as large capacities for harboring analytes or small controlled pores for stronger interactions with the framework. The manipulation of MOF pore size for traditional size-based exclusion filtration can be an effective gas separation technique [17]. Based on this pore topology (e.g., pore shapes, high surface areas, and large volumes), many examples in literature have used MOFs as scaffold materials to further develop superior controlled composites [18,19].

**Figure 3:**
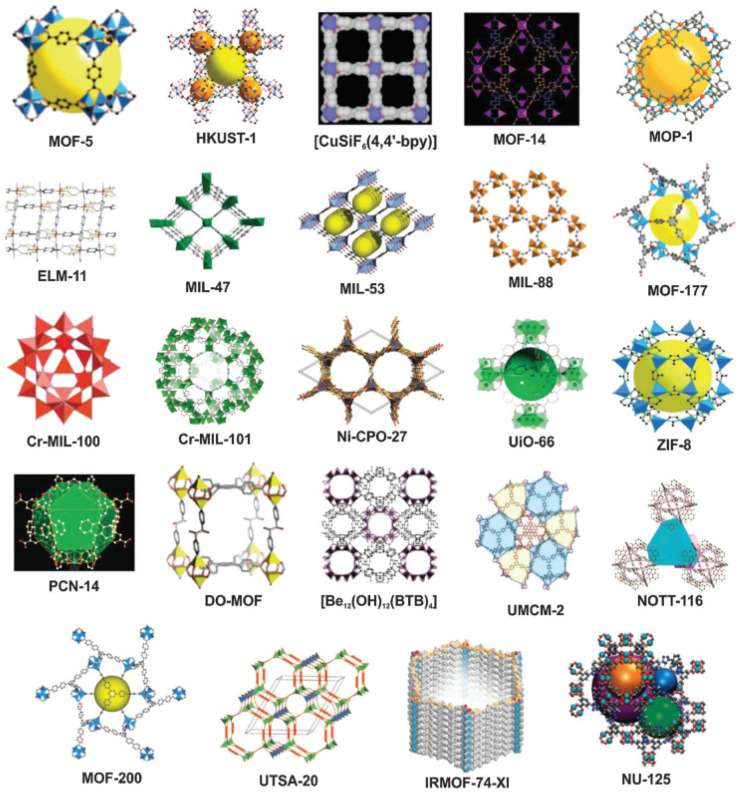
Unit cells showing network topology and void volumes within pores of various representative MOF structures [21].

With thousands of reported ordered structures and even more that continue to be added on a regular basis, it becomes increasingly challenging to use databases that can classify these MOF structures appropriately. There are many ways to classify MOF types, including textural properties such as surface area or pore volume; by subsets of MOF families characterized by similar SBUs, metal centers, or organic linkers; network connectivity for 1D, 2D, or 3D structures; surface functionalities or specific chemical moieties present within the structures; or by application or target analyte uptake performance [20]. Examples of unit cells from MOF structures with varying network topology and shape/size are shown in Figure 3.

**Figure 4:**
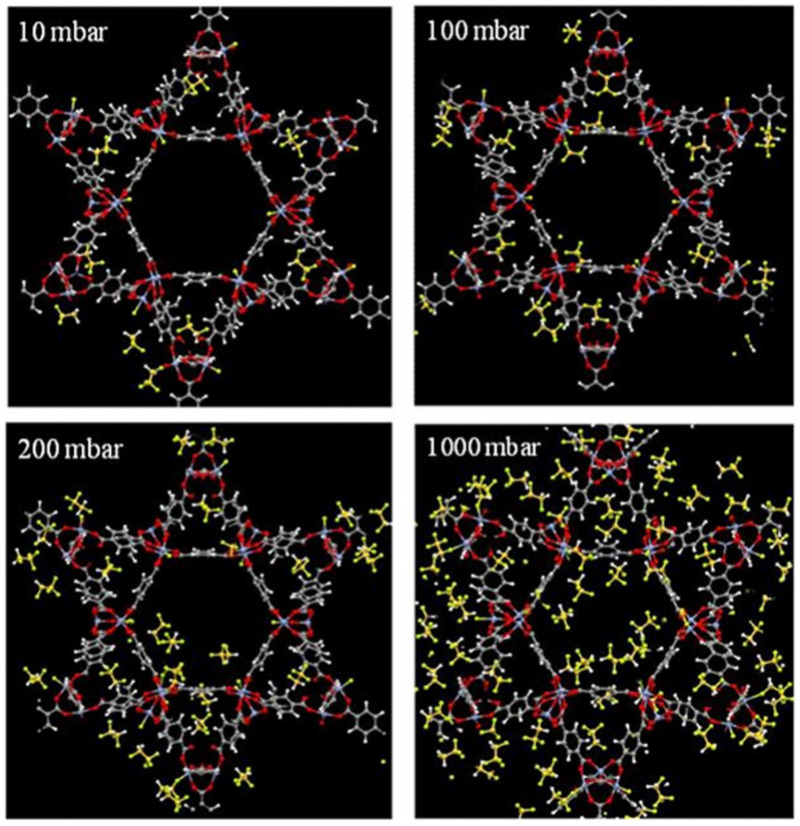
Molecular simulation snapshots showing increasing analyte concentrations within a MOF, revealing both adsorption and guest capture interactions [22].

Apart from the inherent characteristics of the MOF itself, the interaction of the porous host material with a guest molecule either via solvent or in the gas phase can be characterized in many ways, depending on the application. Analyte interactions occurring within MOFs are described in one of two ways, capture or sorption. Capture can be defined by the overall uptake from the bulk phase outside of the MOF into the pore volume of the MOF. Sorption in MOFs can be defined by any analyte interactions with the surface of the MOF pore (adsorption and desorption). At low analyte concentrations, capture and adsorption are synonymous, as it is typically assumed that analytes present in the pore volume will interact with the surface via van der Waals forces or stronger coordination with chemical constituents on the pore surface. At higher analyte concentrations, the capture/uptake of analyte may be high, but there may also be significant analyte–analyte interactions that allow it to “condense” into the pore without interacting with the surface, and hence the adsorption, by definition, is low. Examples of this difference are typically more obvious for larger pore MOFs where localized pore volumes are large enough to fit multiple analyte molecules (as shown in Figure 4). Capture or uptake is typically quantified by analyzing differences in the bulk phase concentration before and after MOF contact via gravimetric or volumetric measurements. On the other hand, sorption is indirectly measured by the energy imparted from surface interaction and can be quantified via calorimetric studies.

**Figure 5:**
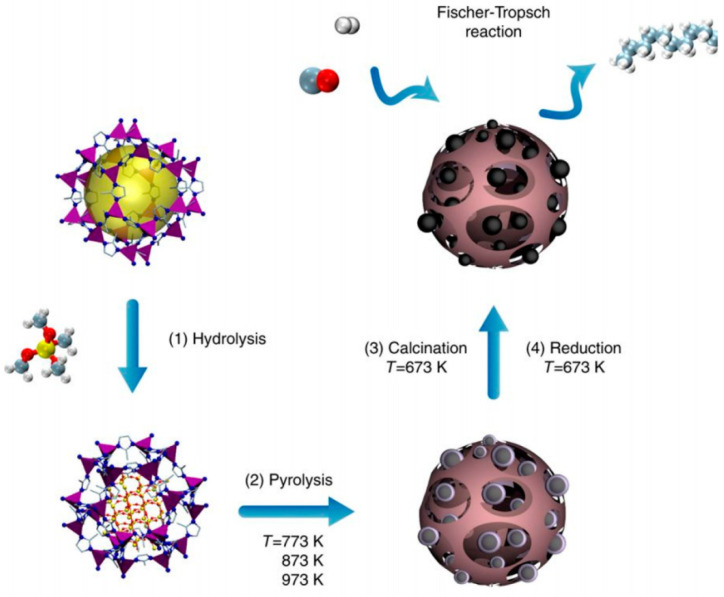
Schematic illustration of catalyst development from MOF precursor via pyrolysis [28].

The high density of metal nodes or other functionalities exposed in the highly porous surface of MOFs is particularly appealing for catalysis where the efficiency of catalyzation is dependent on access to catalyst sites [23]. Compared to traditionally used metal-oxide catalysts that are not porous and tend to agglomerate, the homogenously spread distribution of active sites in these ordered MOFs can be tremendously advantageous [24]. In fact, the use of MOFs as precursors in composites followed by pyrolysis has been shown to be an effective methodology (depicted in Figure 5) to utilize the well distributed density of metal-oxide sites while retaining some level of porosity [24]. To this end, the natural presence of defect sites, defined as coordination sites that were meant to self-crystallize but did not in typical MOF synthesis, have been shown to offer additional high affinity sites similar to the pyrolysis method but without the need to degrade the carbon-based organic linkers and with increased level of control [25,26]. Recent emphasis in MOF defect research has focused on quantifying and synthetically controlling the level of defects using capping agents for coordination sites or mixed linkers for incomplete networking [26]. Many other types of variances in the structure representing the anisotropy introduced in the repetitive backbone framework of MOFs, including mixed metals, mixed linkers, ligand complexation within pores, functionalities, space appropriation, etc., are an emerging field in MOF literature [27].

**Figure 6:**
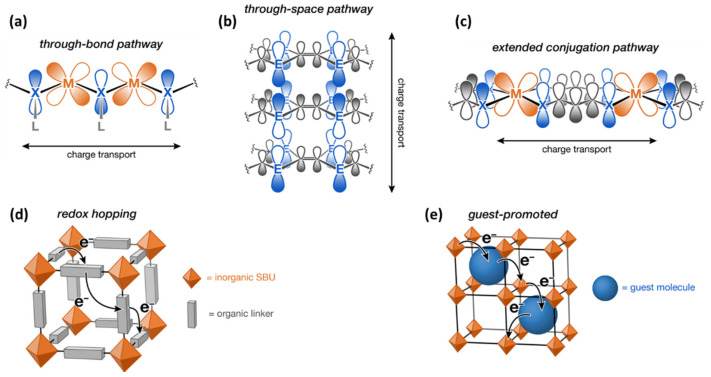
Various mechanisms of charge transport (**a**–**e**) possible in MOFs [29].

In addition to the physical and chemical properties of the framework, limited MOFs have also been studied for their electrical properties. Since MOFs crystallize to form particulates or small grains, the voids between the individual grains typically result in an overall low electrical charge transport, and in general, they are characterized as insulators with low conductivity [29]. However, given the coordination of the framework with strong covalent bonds (versus ionic bonds), more band-like electron transport is possible where valence states of framework constituents are delocalized to facilitate electron shuttling [30]. General strategies for engineering intrinsically electrically conductive MOFs rely on more delocalization between bonds of the framework in combination with a high degree of continuous charge transport pathways [29,31]. The hypothesized mechanisms for electron conduction in MOFs are captured by the schematic shown in Figure 6. These include band like charge transport either through bonds of connected SBUs, through π–π stacking between layers of organic ligands, or through π–d conjugation between connected SBUs and ligands as well as charge hopping transport via redox-active metals-linker or via host–guest interactions [30,31]. Examples of charge delocalization by organic linkers and conductive metal centers include thiolated analogs of MOF-74, Mn_2_(DSBDC)(DMF)_2_ and Fe_2_(DSBDC)(DMF)_2_ [32,33], dithiolene-based 2D MOF, Ni_3_(HITP)_2_, and Cu_3_(HITP)_2_ [34], or (NBu_4_)_2_Fe_2_^3^(DHBQ)_3_ [35] containing a paramagnetic semiquinoid linker, among many others. Increasing conductivity via dopants such as redox-active molecules (tetracyanoquinodimethane, iodine, ferrocene, metallacarboranes, etc.), metallic species/nanoparticles, or other polymers has also been shown [36]. Alternatively, electron transport can also be performed on an entirely different conductive surface, such as a metal oxide or carbon on which the MOF is assembled/adhered, where the role of the MOF is simply to be a medium to promote the shuttling of the charge carrier to the conductive surface [36].

**Figure 7:**
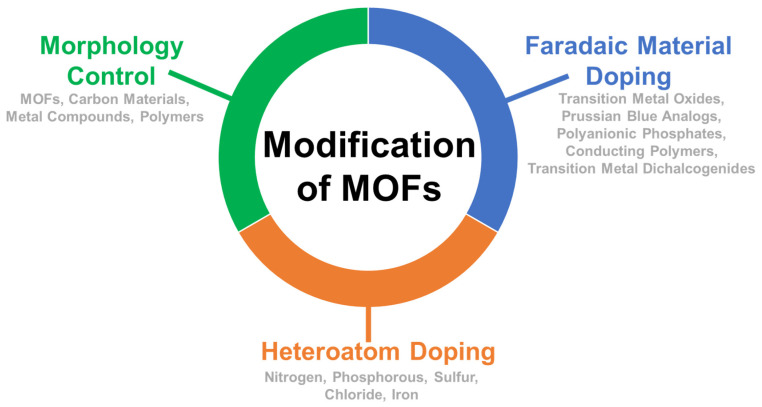
Examples of MOFS modification processes that enhance the electrochemical performance [37].

MOFs traditionally have low electrical conductivity, resulting in electrodes with imperfect electrical properties. Additionally, their apparent high synthesis cost and perceived low stability have hindered their commercial application for industrial processes (e.g., desalination, wastewater treatment, etc.). Recent developments in the carbonization of MOFs (MOFCs) have resulted in the development of materials with improved electrochemical properties compared to traditional MOFs and have addressed some of the shortcomings for electrochemical applications (Figure 7) [37]. For example, templating MOFs with other materials (e.g., carbon, polymer) to control their morphology and structure is an effective way to optimize the MOFs arrangement, increase electrical conductivity, specific capacitance (*SC*), electrosorption capacity (*ESC*), and specific surface area (SA) [37,38,39,40,41,42,43,44,45,46,47,48]. Heteroatom doping has been reported to improve the hydrophilicity and stability of the material, which leads to higher adsorption capacities [37,45,49]. Lastly, Faradic material doping improves the charge adsorption capacity, which yield to higher *SC*, *ESC*, and cycling stability [50,51].

Complementing this understanding of electron transport in MOFs or MOF composites in addition to the mechanism of host–guest interactions during analyte adsorption or capture has helped researchers evaluate these candidates for complex real-world applications. Herein, we focus on strategies for MOF use to benefit electrochemical/electrocatalysis reactions for water treatment.

## 3. MOFS as Indirect Catalysts for Electrochemical Fenton-like Processes

Electrochemical Fenton-based processes are some of the most effective technologies for the degradation of organic pollutants. They improved notably conventional wastewater technologies due to their advantages such as environmental compatibility and high energy efficiency [52]. Electrochemical Fenton-based processes are based on the electrochemical production of hydrogen peroxide (H_2_O_2_) from the cathodic 2-electron oxygen (O_2_) reduction in carbonaceous cathodes in acidic medium via the O_2_ reduction reaction (ORR, Reaction (1)) [53,54,55]. The in situ electrochemical production of H_2_O_2_ can avoid costs and risks associated transport and storage. Depending on the cathodic selectivity, the production of H_2_O_2_ can compete with the reduction of H^+^ to H_2_ and the four reductions in O_2_ to H_2_O [56].

O_2_ + 2H^+^ + 2e^−^ → H_2_O_2_ (1)

**Figure 8:**
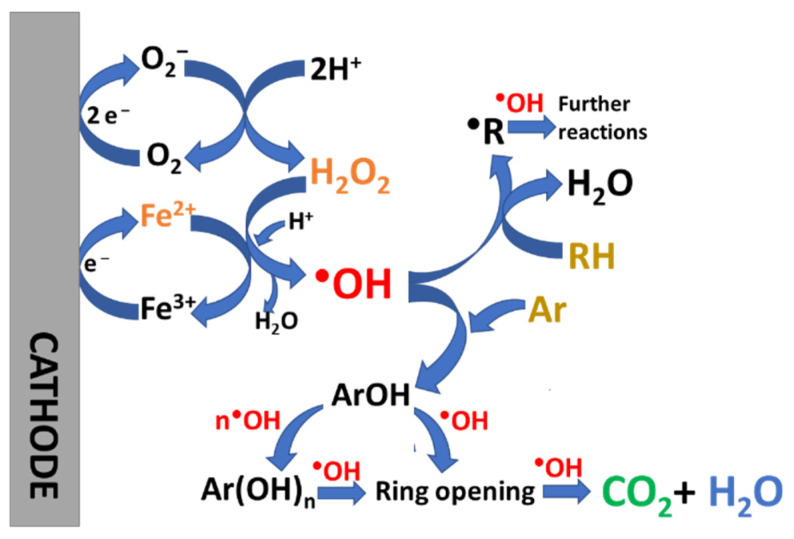
Schematic representation of the main reactions involved in the EF process. The Ar and RH illustrate aromatic and unsaturated compounds that react with hydroxyl radical in the bulk.

Among electrochemical Fenton-based process, homogeneous electro-Fenton process (EF) is the simplest and consists of the external addition of Fe^2+^, while H_2_O_2_ is electrochemically generated in the solution to produce highly oxidative hydroxyl radical (^●^OH) from the well-established Fenton Reaction (2) [57,58]. In EF process, only a small amount of Fe^2+^ is required because of the continuous Fe^2+^ regeneration from Fe^3+^ reduction at the cathode (Reaction (3)), maintaining the continuous production of ^●^OH within the Fe^3+^/Fe^2+^ cycle [59]. The ^●^OH produced from the Fenton reaction can degrade non selectively most organic pollutants to carbon dioxide (CO_2_) and water. A schematic for the EF process can be found in Figure 8.

Fe^2+^ + H_2_O_2_ + H^+^ → ^●^OH + Fe^3+^ + H_2_O (2)

Fe^3+^ + e^−^ → Fe^2+^ (3)

Photoelectro-Fenton (PEF) is another electrochemical Fenton-based process that has received considerable attention as a useful process for the efficient removal of pollutants. PEF consists of simultaneous UV light or solar irradiation of the solution treated under EF to photoexcite Fe^3+^ complexes formed to produce more ^•^OH and regenerate Fe^2+^ (Reaction (4)), as well as the photodecarboxylation of Fe^3+^ with sort chain carboxylic acid (Reaction (5)), thus increasing the efficiency of the process [60,61,62].

Fe(OH)^2+^ + h*v* → Fe^2+^ + ^•^OH (4)

Fe(OOCR)^2+^ + h*v* → Fe^2+^ + CO_2_ + R^•^ (5)

Both electrochemical Fenton-based process are efficient for the complete degradation and mineralization of different class of organic pollutants and promising results are obtained for their future application at industrial level [52]. However, there are some challenges for their commercialization, such as the restricted acid pH range, the loss of catalyst, and the poor catalyst activity and reusability [63]. To overcome these challenges, heterogenous processes using an Fe-based natural or synthetic solid as heterogeneous catalyst have been widely studied in recent years [64,65]. However, most of the proposed heterogeneous catalyst have low catalytic activities and poor stability due to Fe leaching. Therefore, developing effective and durable catalyst remains a challenge. 

Recent advances in nanotechnologies have brought new opportunities in electrochemical remediation of wastewater. In electrochemical Fenton-based processes, the activity, selectivity, and stability of the catalyst are the primary factor affecting the pollutants removal efficiency of the systems [66]. Through nanotechnology, it may be possible to control chemical structure, morphology, and material size to obtain catalyst that could endow some wastewater treatment technologies with exceptional catalytic properties that enhance treatment cost-efficiency. The use of multifunctional advanced materials in electrochemical Fenton-based processes may improve the efficiency of the processes by the combination of multiple treatment functions, such as adsorption, catalytic degradation, and high efficiency of electrochemical H_2_O_2_ generation. The use of engineering nanomaterials such as MOFs and derived materials has caught the attention of many researchers. During the last years, several works have been published in electrochemical Fenton-based processes, exploring the effectiveness of MOF-based nanoparticles (NPs) as Fenton reaction catalysts to remove pollutants from water and improve the efficiency of electrochemical H_2_O_2_ generation [67]. In electro-Fenton systems, the main oxidant is the hydroxyl radical that is generated through the Fenton reaction. The role of MOFs as direct and indirect catalyst of Fenton’s reaction is defined by the involvement in the ^•^OH generation. Most of the use of MOFs in electro-Fenton systems are related to the enhanced synthesis of H_2_O_2_. The electroreduction in oxygen allows the generation of higher amount of H_2_O_2_ more efficiently but does not catalyze the Fenton reaction per se. Therefore, in that case, it is considered an indirect catalyst. In the case where MOFs are used to catalyze the Fenton reaction through Fenton-like mechanisms, the role of the MOF would be defined as direct catalytic effect, since the role of the MOF is directly involved in the generation of ^•^OH.

The electrochemical production of H_2_O_2_ is related to the type and content of heteroatoms that can enhance the electrochemical properties of carbon-based materials [67]. Recently, various MOFs-based electrodes have been explored to modify the cathode material to enhance the production of H_2_O_2_, owing the greater accessibility of active sites for ORR and the reduction in ORR barrier to the presence of an adjoining nitrogen atom [68]. For example, Zhang et al. [69] reported the use of N-containing MOF (zinc pyridine-2,6-dicarboxylate, ZnPDA) as a precursor material for the fabrication of porous carbon materials (i.e., MOFCs). The authors prepared nitrogen-doped porous carbon (NPC) loading carbon paper cathode by direct carbonization of ZnPDA in a tube furnace at various temperatures under N_2_ atmosphere. After calcination, the authors found a foamy and porous structure with uniform distribution of N element in carbon framework at the optimum temperature, which provided high accessibility of active sites for O_2_ reduction and adsorption of reactive, promoting H_2_O_2_ production. High H_2_O_2_ production rate (52.3 mM h^−1^ at −0.5 V, 81.9% efficiency) and selectivity (96.4%) were achieved. These obtained MOFC-based cathode catalysts are used in EF performance for the removal of various organic pollutants, using iron salt as a Fenton catalyst, and the removal efficiency and TOC removal reached 97.9–100% and 71.0–92.0% in 10 and 60 min, respectively (Table 1). Yu et al. [68] also evaluated the modification of an active carbon fiber electrode by zeolitic imidazolate framework-8 (ZIF-8) for efficient H_2_O_2_ production. The modification of ACF by ZIF-8 greatly improved H_2_O_2_ production owing to the porous structure of ZIF-8, which enhanced the reduction in O_2_. Authors found that the amount of ZIF-8 must be optimized, because excessive porous carbon would cause surface crack leading to a decrease in O_2_ reduction and diffusion. They also confirmed with SEM the firm adhesion of ACF to the optimum amount of ZIF-8, which create an adequate number of active sites beneficial to O_2_ diffusion and achieve high H_2_O_2_ generation and efficient degradation of contaminants. Good stability of MOF-based cathodes was demonstrated after various runs (Table 1), indicating that the obtained cathode from MOF is a cost-effective strategy for electrochemical removal of contaminants by Fenton-based processes.

**Figure 9:**
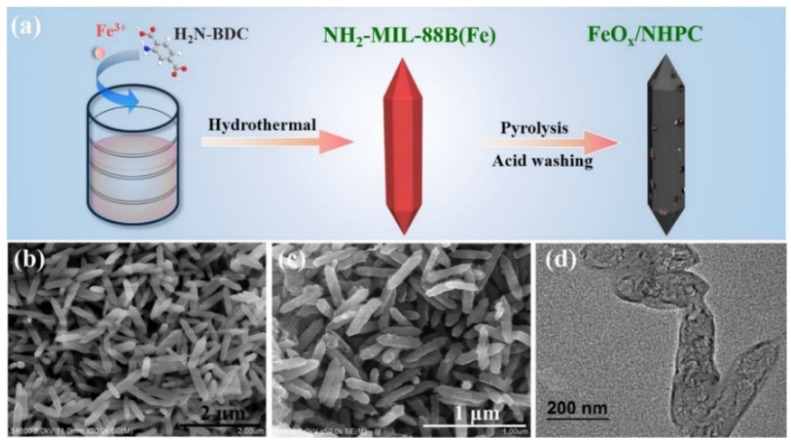
(**a**) Scheme of the fabrication process for FeOx/NHPC; (**b**) SEM image of NH2-MIL-88B(Fe); (**c**) scanning and (**d**) transmission electron microscopy images of FeOx/NHPC750 [76].

Considering the limitations of catalytic activation of H_2_O_2_ to ^●^OH in homogeneous electrochemical Fenton-based processes (e.g., easy precipitation of dissolved Fe (II), pH dependence, catalyst recovery), cathode heterogeneous Fenton catalysts are preferred to overcome these limitations. Hence, the development of dual-functional electro-Fenton catalysts using MOFs for high-yield electrochemical generation of H_2_O_2_ and in situ activation of H_2_O_2_ to ^●^OH has attracted tremendous attention. For example, the introduction of Fe-based materials into carbon materials can enhance the conductivity and enhance the reduction of Fe^3+^ to Fe^2+^; however, the presence of metal in the carbon-based electrode can affect the ORR from a 2 to a 4 e^−^ process [70]. Moreover, the periodic arrangement of metal nodes and the coordination ligands of MOFs can increase the accessibility and oxidation level of Fe atoms [71]. The development of these materials may allow one to overcome the mentioned limitations of homogeneous and heterogeneous electrochemical Fenton-based processes. For example, Zhang et al. [72] evaluated the use of a hollow sea-urchin-shaped carbon-anchored single-atom Fe (SAFe_x_@HSC) derived from MOFs as a dual functional EF catalyst. The electrochemical production of H_2_O_2_ using SAFe_x_@HSC derived from MOF showed excellent OER with an improvement in activity and selectivity toward two electrons reduction, which produced H_2_O_2_. This finding opens further avenues for the optimization and application of MOFs based on indirect electrochemical Fenton-based processes. The activation of H_2_O_2_ to ^●^OH in the SAFe_x_@HSC system was confirmed by electron spin resonance spectroscopy, and the ^●^OH concentration improved by increasing Fe content in SAFe_x_@HSC. The effective activation of H_2_O_2_ was demonstrated by the high Thiamphenicol removal efficiency (100%) in a broad pH range, indicating the positive impact of an MOF-based electrode for the application of indirect electrochemical Fenton-based processes. The stability of the catalyst was confirmed by continuous flow and reusability of electrode for six cycles. Xiao et al. [73] performed the heterogeneous EF reaction using N-doped graphitic-carbon-coated Fe composite dispersed in a N-doped carbon framework (Fe_3_N@NG/NC) as a cathode for the electrochemical H_2_O_2_ generation, and their activation to ^●^OH. Fe_3_N@NG/NC was obtained by carbonization and ammonia etching of the MOF MIL-101 (Fe) producing well dispersed N-doped carbon-coated Fe_3_N NPs. A high removal (100%) rate of various dyes (e.g., Rhodamine B, dimethyl phthalate, Methylene blue, and Orange II) was obtained at a wide pH range (3.0 to 9.0) with very low Fe leaching (<0.03 mg/L), demonstrating the important role of MOFs to overcome abovementioned limitations of Electrochemical Fenton-based processes. The cathode developed by Xiao et al. maintained high removal efficiency after five cycles of reutilization, indicating that the cathode can be used for cost-effective removal of organic pollutants by EF process. Lu et al. [74] used the MOFs MIL100-(Fe) and MIL-53(Fe) to manufacture a porous composite of ferric oxide/nitrogen/carbon (Fe_2_O_3_/N−C) cathode for efficient production of H_2_O_2_. The prepared cathode showed good ORR activity for H_2_O_2_ production confirming the positive contribution of MOFs for the 2-electron ORR pathway beneficial for the electrochemical processes based on the Fenton reaction. The incorporated hematite allowed activation of H_2_O_2_ for the efficient degradation of bisphenol A and other contaminants by heterogeneous EF process (Table 1). The authors found that the precursor MIL-100(Fe) is more suitable than MIL-53(Fe) due to low content of C-N bond of MIL-53(Fe) and small specific surface area. Liu et al. [75] used different MOFs as precursors to study the impact of MOFs topology and functional group on the subsequent electrocatalytic properties of the cathode. The results showed that the narrow pore distributions allowed high electrochemical H_2_O_2_ productions and facile access to the active metal catalytic centers. In the study carried out by Cao et al. [76], N-doped hierarchically porous carbon with embedded FeOx (FeOx/NHPC) was designed to be used as a bifunctional catalyst for the effective removal of pollutants. The bifunctional catalyst FeOx/NHPC was prepared by one-step carbonization of and Fe-based MOF (NH2-MIL-88B(Fe)), as illustrated in Figure 9a. After carbonization at 750 °C, NPs with bipyramidal hexagonal prism form similar to NH2-MIL-88B(Fe) were obtained (Figure 9b,c). The porous structure of FeOx/NHPC was observed via TEM analysis. FeOx/NHPC obtained were also found active as two electrons ORR with high selectivity. The increase in selectivity using MOF improved the production of desirable products and the energy utilization for future application of electrochemical Fenton-based processes at industrial level.

**Figure 10:**
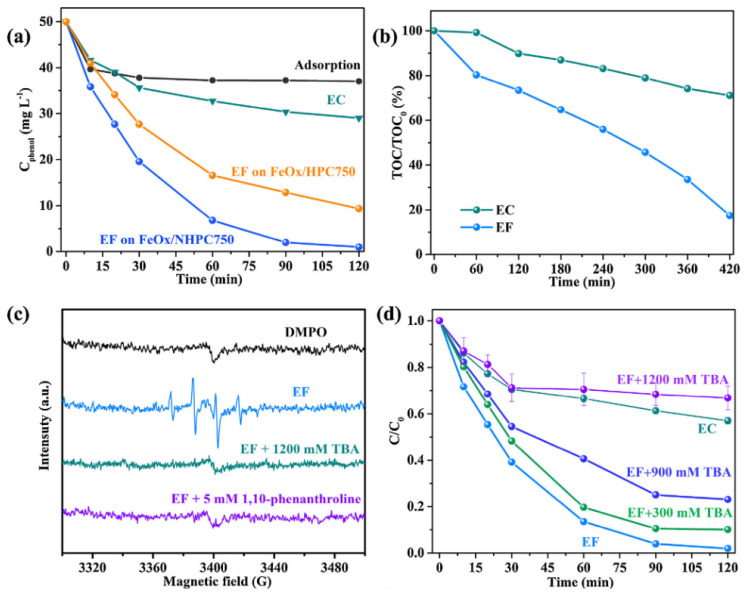
(**a**) Phenol removal curves of EC and hetero-EF on FeOx/NHPC750, as well as hetero-EF on FeOx/HPC750 at −0.6 V; (**b**) TOC removal curves for EC and hetero-EF on FeOx/NHPC750; (**c**) DMPO spin-trapping spin resonance spectroscopy spectra during FeOx/NHPC750 EF process with 1500 mM TBA or 5 mM 1,10-phenanthroline; (**d**) effect of TBA concentration on hetero-EF degradation of phenol [76].

The effectiveness of FeOx/NHPC as a catalyst in EF process was confirmed by almost complete removal (98%) of phenol and 83% TOC removal after 120 min electrolysis (Figure 10a,b). The authors demonstrated the effective activation of H_2_O_2_ to produce ^●^OH by spin trapping for spin resonance spectroscopy using DMPO in the presence and absence of ^●^OH scavenger (Figure 10c,d). The increase in H_2_O_2_ product selectivity and subsequent activation into ^●^OH is related to the N configuration and content, which improve electron transfer to accelerate ORR and Fe(III) reduction. Dong et al. [77] developed an efficient and robust cathode using MIL-101(Fe) as a precursor for the electrodegradation of p-nitrophenol. The authors prepared a CFP@PANI@MIL-101(400) cathode by controlled pyrolysis of the MIL-101(Fe)-based polyaniline (PANI) modified carbon fiber papers (CFP) at 400 °C. Their results showed excellent catalytic activity and high stability for the removal of pollutants in electrochemical Fenton-based processes, achieving high H_2_O_2_ production (200 mg L^−1^ H_2_O_2_ after 120 min), complete removal of p-nitrophenol, and 52% TOC removal. The enhanced results were explained by the synergistic effects among the accessible Fe-O sites, formation of Fe_3_O_4_ NPs, and encapsulation by graphene-like carbon layer. Cao et al. [78] designed similar cathode using NH_2_-MIL-88B(Fe) and incorporating Cu as co-metal to promote Fe(II) regeneration, which greatly promoted in situ activation of H_2_O_2_ to produce ^●^OH. The FeOx/CuNxHPC cathode exhibited excellent phenol degradation and mineralization abilities at pH 4–10. Similarly, Qiu et al. [79] fabricated a bimetallic cathode using Ce as a co-catalyst, which increased the electrocatalytic activity of the electrodes (100% removal of Sulfametoxazole in 120 min). Wang et al. [80] reported the use Cu-based MOF for the synthesis of MOF derived embedded in N-doped carbon composite cathode (Cu/N-C) in EF process. The Cu/N-C catalyst showed high electrocatalytic activity for H_2_O_2_ production and activation, leading to efficient removal of bisphenol A (Table 1). In summary, these MOF-derived electrodes are beneficial for continuous operation, as they demonstrate stability after reusing due the low metal leaching and no agglomeration of metal NPs. 

On the other hand, MOF-derived materials can also be used alone as suspended heterogenous catalyst in the solution to activate H_2_O_2_ in EF process. Du et al. [81] prepared a magnetic NP (Fe/Fe_3_C@PC) using MIL-101(Fe) as precursor by a simple pyrolysis method. EF process was carried out using Fe/Fe_3_C@PC as heterogeneous catalyst for the removal of sulfamethazine. His results showed that Fe/Fe_3_C@PC outperformed common heterogeneous catalyst such as Fe^0^, Fe_3_O_4_ and Fe_2_O_3_. Ye et al. [82] reported the preparation of pyrite NPs linked to porous carbon (Fe_2_S/C) by simultaneous carbonization and sulfidation of Fe-based MOF. Efficient removal of fluoxetine was observed by authors and the enhancement of catalytic activity was confirmed by comparing with natural pyrite and Fe salt. Ye et al. [83] also evaluated the use of nano-ZVI@C and nano-ZVI@C-N obtained from MIL(Fe)-88B and NH_2_-MIL(Fe)-88B, respectively, to evaluate the effect of functional group. The nano-ZVI@C-N showed highest electrolytic activity with superior pollutant removal (57 and 90% removal after 60 min for ZVI@C and ZVI@C-N, respectively). This excellent performance of suspended MOF-based catalysts may be attributed to the wide catalytic surface area and rich active sites. On the other hand, effective separation of catalyst after treatment is achieved by virtue of magnetic property of synthetized MOFs using an external magnetic field to overcome the limitation of catalyst reutilization.

A very effective way to improve the previous studied heterogeneous EF is coupling with UV or solar radiation in the PEF process. This process favors the production of more ^●^OH and regeneration of catalyst. Furthermore, using a heterogenous catalyst with photocatalytic properties, the process can be upgraded by the contribution of a photogenerated electron-hole to produce more oxidants and enhance the regeneration of the catalyst. For example, Zhao et al. [84] reported the use of bifunctional MOF(2Fe/Co)CA cathode with high photocatalytic activity in solar photoelectron-Fenton for the degradation of Rhodamine B and dimethyl phthalate. MOF(2Fe/Co)CA with photocatalytic and electrocatalytic activities enhanced the performance of PEF-promoting 2 electron pathway ORR and the additional activation of H_2_O_2_ to ^●^OH by photoinduced electron on the conductive band. Ye et al. [85] evaluated the performance of suspended Fe-based 2D MOF as a heterogeneous catalytic in PEF process under illumination with UV or visible light. Heterogeneous PEF was used with an Fe-based 2D MOF electrochemical oxidation process and homogeneous EF and PEF. Efficient activation of H_2_O_2_ to ^●^OH was continuously sustained with Fe^2+^ regeneration from Fe^3+^ reduction by photoinduced electrons, which led to a high degradation efficiency (Table 1). 

## 4. Photoelectrocatalytic Applications of MOFs in Environmental Remediation

**Figure 11:**
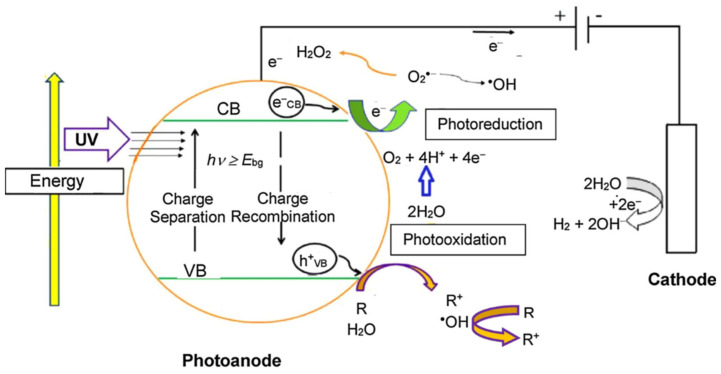
Schematic of the PEC process [49].

The catalytic response of semiconductor materials exposed to light irradiation was first described in the seminal work of Fujishima and Honda in 1972. The photocatalytic effect is observed when a semiconductor is irradiated with photons of energy superior to the characteristic bandgap energy (*E_g_*) of the material [86,87]. The light–semiconductor interaction induces the photoexcitation of an electron from the completely filled valance band (VB) of the semiconductor to the empty conduction band (CB). The photoexcited electron (e_CB_^−^) leaves a reactive vacancy or hole in the valence band (h_VB_^+^), according to Reaction (6) [88]. The e_CB_^−^-and the h_VB_^+^ are commonly referred to as charge carriers, since they are responsible for semiconductor conductance [89]. The e_CB_^−^ is highly reductant, and h_VB_^+^ is a strong oxidant [90]. Furthermore, both species can generate reactive oxygen species in an aqueous solution such as superoxide (O_2_^●−^) from dissolved oxygen reduction by Reaction (7) and hydroxyl radical from water oxidation by Reaction (8) [90]. The high reactivity of both charge carriers can be exploited in redox chemistry applied to the environment, such as water splitting by Reaction (9) [91,92], organic pollutants (R) oxidation according to general expression (10) [93,94], metal pollutants (M) reduction by Reaction (11) [95,96], CO_2_ reduction following Reactions (12)–(14) [97,98], etc. A schematic of the photoelectrocatalytic degradation process can be found in Figure 11.

Semiconductor + *hν* → e_CB_^−^ + h_VB_^+^ (6)

e_CB_^−^ + O_2_ → O_2_^●−^ (7)

h_VB_^+^ + H_2_O → ^●^OH + H^+^ (8)

2 H_2_O → 2 H_2_ + O_2_ (9)

R + h_VB_^+^/^●^OH → x CO_2_ + y H_2_O + inorganic ions (10)

M^x+^ + x e_CB_^−^ → M (11)

CO_2_ + 2H^+^ + 2e^−^ → CO + H_2_O (12)

CO_2_ + 2H^+^ + 2e^−^ → HCOOH (13)

CO_2_ + 8H^+^ + 8e^−^ → CH_4_ + H_2_O (14)

It is important to remark that if the generated charge carriers are under an excited state, then they tend to return to their ground state following their recombination (Reaction (9)) [99]. Different strategies have been explored to slow down the extent of recombination reaction, such as semiconductor doping, semiconductor nano-enabler with electron sinks, or z-schemes. Photoelectrocatalysis (PEC) is one of the most promising approaches, since it not only inhibits the recombination Reaction (15) but also enables the use of immobilized photocatalyst material [100]. The removal/recovery of nanoparticulated semiconductor material is one of the most costly and greater challenges of conventional photocatalytic systems [101]. The application of a bias potential or bias current enables effective charge carrier separation induced by an electrical field, which maximizes the generation of ROS [100,102]. Experimental results demonstrate synergistic effects in PEC derived from the electrification of conventional heterogeneous photocatalytic systems. PEC processes have mostly studied the use of titanium dioxide and other semiconductor materials, such as ZnO, NbO_2_, WO_3_, and mixed metal oxides [103,104]. However, MOF-based photoelectrodes are slowly opening a new path for a third-generation material in PEC systems.

e_CB_^−^ + h_VB_^+^ → heat (15)

As discussed previously, MOFs are molecules arranged in crystalline-like lattices and present exciting properties for several applications. Indeed, some MOFs have been reported to display semiconductor-like behavior through metal-oxo cluster excitation processes [105,106]. It is argued that the metal-oxo clusters and organic linkers can be regarded as isolated semiconductor quantum dots and light-absorbing antenna, respectively [105]. Thereby, MOF photocatalysts present similar electron transitions to bulk semiconductors [107]. However, in MOFs, the electron is photoexcited from the highest occupied molecular orbital (HOMO) to the lowest unoccupied molecular orbital (LUMO) in MOFs, generating holes (h^+^) in the HOMO [105]. Thus, the LUMO/HOMO play an analogic role than the CB/VB, and the charge carriers generate ROS following Reactions (7) and (8). As metal-oxo clusters increase in size towards NP size, the combination of an increasing number of atomic orbitals expands the number of available energy states while pushing the HOMO and LUMO closer together (similar to a band structure) [106]. This effect has been reported to decrease the band gap energy of MOF photocatalysts. 

**Figure 12:**
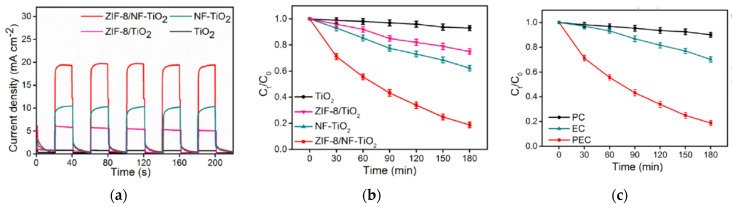
Comparative behavior of pristine TiO_2_, NF-doped TiO_2_, ZIF-8 modified TiO_2_, and ZIF-8 modified NF-TiO_2_**.** (**a**) Photocurrent response, (**b**) photoelectrocatalytic abatement of sulfamtheoxazole by PEC, (**c**) comparative performance between photocatalysis (PC), electrocatalysis (EC), and PEC. Adapted from [109].

Despite the increasing number of publications on photocatalytic MOFs in the literature, photoelectrocatalytic approaches have been seldomly explored. It is true that there are still major research questions regarding the long-term stability of MOF photocatalysts, especially considering that the organic ligands may be susceptible to oxidation/degradation by hydroxyl radical. However, the major barrier is the conductivity of MOFs when embedded in photoelectrodes. Thus, it is not surprising that the main advances on PEC have been conducted on the study of MOF-derived photoelectrodes synthesized from MOF calcination under N_2_ atmosphere [108]. Figure 12a depicts the enhancement of photocurrent response when bare TiO_2_ is modified with a calcined zeolitic imidazolate framework (ZIF) that also makes the composite material light visible active. The application of the ZIF-8/NF-TiO_2_ photoelectrode proposed by Jia et al. [109] demonstrated high PEC activity on the degradation of sulfamethoxazole with an increase of two orders of magnitude in degradation kinetics rate, from a *k*_1_ of 4 × 10^−4^ min^−1^ for TiO_2_ up to 8.9 × 10^−2^ min^−1^ for ZIF-8/NF-TiO_2_ (see Figure 12b). Experimental results evidenced that the electrification results in a synergistic enhancement given the combination of effects from electrochemically driven processes with photo-assisted catalysis as shown in Figure 12c. Thus, the use of a MOF-derived photoelectrode shows increased degradation performance due to the unique MOFs features. 

Similar approaches have been reported on the use of MOF-based structures resulting from their calcination in inert atmosphere. Song et al. showed similar synergistic enhancement when exploiting a combination of ZIF-based structure with TiO_2_ nanotubes to degrade Rhodamine B dye through PEC [110]. Meanwhile, other works have explored the combination of bulk semiconductor material with Fe-based MOFs, carbonized to form heterostructures that are photoactive [111]. The identification of stable organic ligands with electric conductance may become a paradigm shift on novel MOF-based photoelectrodes. Lessons learned during the study of conductive polymers (e.g., polyaniline, polypyrrole) may be used as a guideline to identify promising ligands to synthesize pure MOF structures with photoelectrocatalytic capabilities. However, prior to the identification of such nanoengineered structures, there are fundamental questions to be answered through the use of carbonized MOF materials.

## 5. Selective Adsorption Capabilities of MOFs Induced by Non-Faradaic Processes

**Figure 13:**
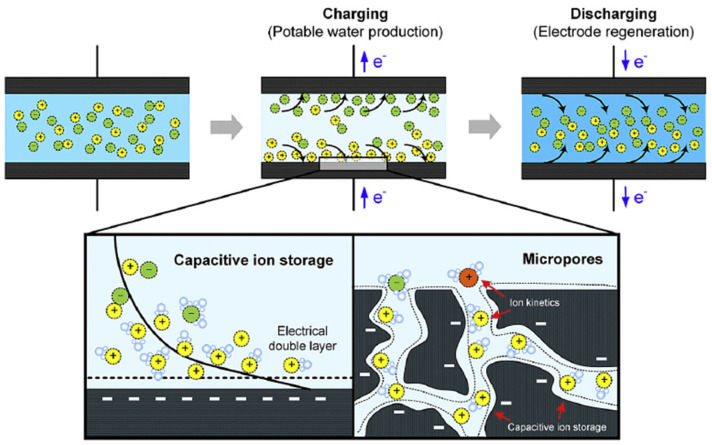
Diagram of capacitive deionization (CDI) operation and non-Faradic CDI processes [114].

Capacitive deionization (CDI) is a novel technology used to remove ions from brine and brackish waters and generate fresh water. A CDI system consists of a pair of porous electrodes (anode and cathode) separated via an open channel (where the solution flows) or a dielectric material. Both electrodes are charged with an applied voltage, causing salt ions to migrate from the solution into the electrical double layers (EDLs) along the pore surfaces at the electrode/solution interphase, generating clean fresh water. Once the surface of the electrodes is saturated with the salt ions, a discharge step is used by stopping or reversing the applied potential to release the salt ions into a brine stream and regenerate the sorbent (Figure 13) [112,113,114,115].

**Figure 14:**
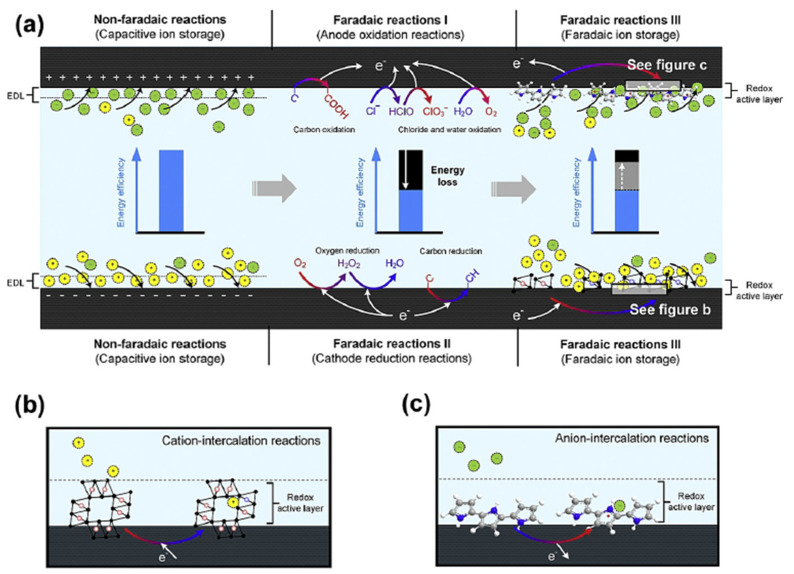
(**a**–**c**)Schematic presentation of three types of faradaic processes (anodic oxidation, cathodic reduction, and faradaic ion storage processes) [114].

The CDI electrodes can be categorized into non-faradaic electrodes and faradaic electrodes according to the ion adsorption mechanism [37,113,114,116]. Most carbon-based CDI processes are non-faradaic, in which ions are stored in the EDLs formed within the pore structure of the electrode, and no reactions/transformations are involved. However, faradaic electrodes involving redox reactions (e.g., anodic oxidations and cathodic reduction) are also utilized to store ions mainly based on the faradaic reaction, which have attracted wide attention for their typical high electrosorption capacities and cycling stability [37,113,114]. A summary of the different CDI processes is shown in Figure 14. Because the faradaic processes are associated with (i) the degradation of carbon-based electrodes (e.g., via oxidation of the electrode), (ii) the formation of undesired side products (i.e., CO_2_, H_2_, O_2_, H_2_O_2_, HCl, etc.), and (iii) a lower energy efficiency [114,116,117,118], they are not considered in this section. Instead, this section will focus on discussing the non-faradaic process.

Among the different factors affecting the CDI performance of non-faradaic processes (e.g., device, operation conditions, water composition) [38,112,118,119,120,121], the sorbent properties such as (i) electrosorption capacity (*ESC*), (ii) stability, and (iii) charge efficiency (CE) are the most crucial and determine the feasibility of the process. For example, *ESC* determines the total amount of sorbent needed to remove the species form the wastewater, which will impact the size of the system and how often the sorbent material needs to be replaced. Similarly, the stability of the sorbent determines how often it needs to be replaced and any treatment required to regenerate the sorbent. The charge efficiency (CE) determines the amount of current consumed in the target reaction instead of side and faradaic reactions. The lower the CE of the CDI system, the higher the generation of this side products and the cost of the processes required to manage them. For example, Liu et al., reported that 45% of the CDI cost is associated with electrode cost [120], i.e., capital and replacement, and 34% are associated with electricity cost. Hence, finding sorbent materials with high *ESC*, stability, and CE is paramount to the feasibility of the non-faradaic CDI process and its cost-competitiveness against contending technologies, such as reverse osmosis.

Besides *ESC*, the other main properties of CDI materials are electrosorption rate (*ESR*), specific surface area (*SA*), and specific capacitance (*SC*). The SA is traditionally obtained via N_2_ Brunauer–Emmett–Teller (BET). *ESC* is obtained by comparing the concentration of species (e.g., NaCl) in solution before and after CDI normalized by the electrode mass, Equation (16). *ESR* is obtained by normalizing *ESC* by the length of time CDI was performed (Equation (17)). *SC* is used to quantify the electrode capacity to store energy (i.e., anions and cations) and is obtained from cyclic voltammetry (CV) curves (Equation (18)). The CDI electrical efficiency of different materials is assessed via the CE, which is the ratio between the *ESC* and charge (Equation (19)). The overall performance of the CDI system is assessed via the removal efficiency (RE), which is the ratio between the concentrations before and after the removal (Equation (20)).

(16) $$ESC\;\left(\mathrm{mg}/g\right)=\frac{\left(C_{0}-C_{t}\right)\cdot V}{m}$$

where *C*_0_ is the initial concentration of species (mg/L), *C_t_* is the concentration of species after CDI (mg/L) for a particular length of time, *V* is the solution volume (L), and *m* is the mass of the electrode (g).

(17) ESR (mgg·h)=ESCt

where *ESC* is the total mass of species removed normalized by the mass of the electrode in a particular length of time (mg/g), and *t* is the length of time (h).

(18) $$SC\;\left(F/g\right)=\frac{\int idV}{m\cdot \Delta V\cdot S}$$

where *i* is the current (A), *m* is the mass of the electrode (g), ∆*V* is the potential window (V), and *S* is the scan rate (mV/s).

(19) $$CE\;\left(\%\right)=\frac{ESC\cdot F\cdot m}{C}\times 100$$

where *ESC* is the electrosorption capacity (mg/g), *F* is the Faraday constant (96,485 C/mol), *m* is the mass of the electrode (g), and *C* is the charge (C) obtained by integrating the corresponding current (A) as a function of time (s) used in the CDI experiment.

(20) $$RE\;\left(\%\right)=\frac{\left(C_{0}-C_{t}\right)}{C_{t}}\times 100$$

where *C*_0_ is the initial concentration (mg/L), and *C_t_* is the concentration after CDI (mg/L) for a particular length of time.

Traditional CDI electrodes are carbon-based, such as active carbon (AC), hierarchically porous carbon (HPC), carbon nanotubes (CNT), and graphene (GE). A summary of the properties of this different class of materials can be found in Table 2, adapted from Zhao et al. [112]. Carbon-based materials tend to be favored for CDI applications, as they are low cost and have desirable *SC* and *ESC* properties, which are correlated with the *SA*. As illustrated in Table 3, the CDI performance (i.e., RE) of these materials are affected by reaction conditions and operation mode for traditional AC. Hence, when evaluating new CDI materials, the researchers are recommended to baseline their performance against traditional materials (such as AC, HPC); however, if not possible, the researchers should compare their results against literature values obtained under similar reaction conditions and apparatus.

Another class of materials that has been gaining interest in the CDI field are MOFs, due to their designable framework structures, tunable surface properties, and facile fabrication [37,39,41,45,46,47,48]. Recent developments in MOFCs have resulted in the development of materials with seemingly similar CDI properties to that of traditional carbons (Table 3). 

**Figure 15:**
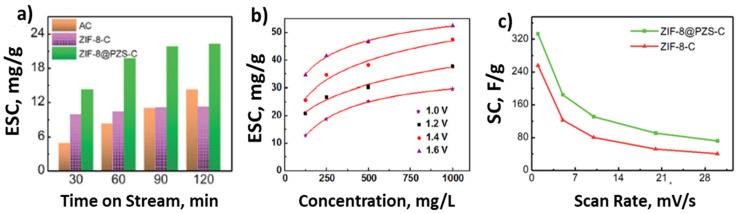
(**a**) Plot of *SC* vs. deionization time of stream of various electrodes in a 500 mg/L NaCl solution with flow rates of 50 mL/min. (**b**) The electrosorption isotherms of PC/rGO-20 electrode as a function of cell potentials and NaCl concentration. (**c**) Specific capacitance of ZIF-8-C and ZIF-8@PZS-C at different scan rates. Adapted from [49,137].

The CDI-related properties (i.e., *ESC*, *SC*) of the new MOFC formulations have reportedly achieved and exceeded those of traditional carbons (i.e., AC, HPC). However, the readers should note that the experimental reaction conditions (e.g., potential, concentration, experiment length) as well as reaction apparatus (e.g., batch vs. continuous flow, distance between electrodes, electrode surface area) affect the CDI properties of the materials [37,38,42,113]; hence, it is not advisable to perform a direct comparison of literature values to quantify improvement, and researchers should baseline the performance of their materials against that of commercial CDI materials (e.g., AC). For example, Zhang et al. showed the performance AC against Zeolitic imidazolate framework (ZIF)-derived materials as a function of time on stream (Figure 15a) [49]. While the *SC* of AC was nearly half that of a carbonized ZIF-8 (ZIF-8-C) during the initial 30 min of reaction, the AC after 120 min was 20% higher than that of ZIF-8C. Similarly, Shi et al., showed that the CDI performance of carbonized Fe-MOF varies with NaCl concentration on solution and operation potential (Figure 15b) [137]. Additionally, the *SC* of CDI electrodes decreases by nearly one order of magnitude when increasing the scan rate from 1 to 20 mV/s (Figure 15c) [49]. Hence, due to the described effects of reaction conditions on CDI performance, we will focus on discussing CDI literature that evaluated their MOFCs under similar conditions and baselined the performances against that of commercial electrodes. The results are summarized in Table 4.

For example, Chang et al. improved the morphology of a MOF-5 via decarbonization (MOF-5-PC) by including a porous carbon-like structure. The performance of MOF-5 for desalination via CDI had 2.3 times higher specific capacity (108 and 48 F/g, respectively) and 70% higher SAC (9.4 and 5.5 mg/g, respectively) than AC [41]. Wang et al., evaluated the effect of using N as a doping material and Faradic materials, such as Zn and Co, on the CDI desalination performance [50]. His results show that the addition of monometallic Zn and Co metals increased the *SC* by a factor of two compared to AC, while the incorporation of both Zn and Co increase the *SC* by 2.7 times compared to AC [50]. Shi et al. reported the performance of Fe-MOF on a hierarchically porous carbon composite with a dimensional graphene oxide network (Fe-MOF-HPC-GO) eight times higher *SC* than that of AC (37.6 and 5.4 mg/g) and showed *SC* with one seven times higher that of AC at similar conditions [137].

ZIFs are a class of MOFs that combine organic and metal frameworks and have also been widely evaluated for CDI applications [40,48,49,50]. For example, Liu et al. synthesized porous carbon polyhedral ZIF (ZIF-8-PCP) and modified its morphology by calcining at different temperatures. In their work, they show that increasing the synthesis temperature from 800 to 1200 °C improved the SA by a factor of two while improving the *ESC* only by 30% [138]. Similarly, Wang et al. evaluated the effect of morphology improvements by calcining ZIF-8-derived carbon at 900 °C, which resulted in an improvement in *SA*, *ESC* and *SC* of 10%, 53%, and 25%, respectively [123]. Zhang et al., reported on the effect of carbonizing ZIF-8 (ZIF-8-C) and N, P, S heteroatom co-doping (ZIF-8@PZS-C) and showed that the *ESC* of ZIF-8-C and ZIF-8@PZS-C nearly doubled and tripled with respect to that of AC [49]. Wang et al. demonstrated that the carbonization of Zn-containing MOFs such as ZIF-8, RT-MOF-5, and ZnFumarate resulted in the formation of MOFCs (ZICarbon, RMCarbon and ZFCarbon, respectively) with ≈3 times higher *ESR* and *ESC* than that of a commercial AC, which were proportional to the increase in *SA* [48]. Shen et al. reported on the carbonization of ZIF-8 to form hollow and solid nanoporous carbons (SZCs and HZCs respective) with nearly one order of magnitude higher *ESR* and three times higher *ESC* than that of commercial AC under identical reaction conditions [48].

In summary, the works reviewed here illustrate how the MOF modifications have improved their properties for CDI applications, making them competitive with traditional carbon materials. However, more work needs to be done to demonstrate their performance and stability under industrially relevant applications (e.g., real brine or wastewater, long-term operation).

## 6. Perspectives and Key Insights on the Future of MOFs

The utilization of MOFs in faradaic (i.e., electro- and photo-electrochemical degradations) and non-faradaic (e.g., CDI) processes has expanded thanks to the recent improvements in the synthesis and modifications of MOFCs. Morphology control, heteroatom doping, and faradaic material doping represents the main modification by which the electro- and photoelectron-chemical properties (e.g., degradation rate, stability, mitigation of side reactions) of MOFCs have improved by nearly one order of magnitude. In particular, the faradaic Fenton-related process such as electro-and photoelectro-Fenton process have benefited the most as the encapsulation the Fenton agent (e.g., Fe, Cu) inside the MOF structure has mitigated the main shortcomings of the EF and PEF process (e.g., metal leaching, and metal recovery). The utilization of MOFCs in non-faradaic CDI applications has also resulted in performance enhancements that exceeded that of traditional (non-)faradaic materials such as carbon-based electrodes.

Since the MOFCs utilization in (non-)faradaic process has already been successfully demonstrated in ideal, lab-scale systems, the next steps should focus on exploring the long-term stability with real feedstocks (i.e., brine and brackish water) and assess their integrity under industrially relevant conditions (e.g., current, potential, temperature, flow rates, and operation mode). In particular, we recommend using physicochemical (X-ray diffraction, surface area, scanning and transmission electron microcopy, X-ray photoelectron spectroscopy) and electrochemical (CV, double layer capacitance, electrochemical impedance spectroscopy) characterization techniques to assess MOF stability and identify the nature of the possible changes in performance as a function of time of stream and cycles. Once long-term stability has been evaluated, the process parameters (i.e., performance, material lifetime, regeneration and replacement, power consumption, energy efficiency) should be used in life-cycle and techno-economic analyses to understand the process economics and ascertain its cost-competitiveness against current commercially used electrodes for contaminant removal and degradation processes.

The highly organized structure derived from MOF materials (with and without calcination) introduce unique nanostructured effects that are essential for enhanced selective electroseparations as well as physical properties related to the electrosorption capacities of the materials. In a similar fashion, structure and homogeneous dispersion of metallic centers have shown outstanding catalytic responses for enhanced electrogeneration of H_2_O_2_. The distribution of metallic catalysts in carbon structures may open new avenues for heterogeneous catalyst stable impregnation/attachment on electrodes to enhance Fenton catalysis even at circumneutral pH. The understanding of the semiconductor properties of MOF-based materials and hierarchically organized structures at molecular level are barely explored areas for improved photoelectrocatalytic applications in the environment that hold the promise to revolutionize efficient light usage and conversion rates. Urgent questions regarding heteroatomic metallic centers of pristine and calcined MOFs can provide a deeper understanding and expand horizon opportunities for electrochemical environmental applications.

## Figures and Tables

**Table 1:** Comparison of heterogeneous EF and PEF performance using MOF-based materials for the treatments of several organic pollutants in different aqueous matrices.

MOFs	Contaminants	Initial Concentration (mg L^−1^)	Cathode/Catalyst	Applied Current or Potential	Removal Efficiency/%	Treatment Time (min)	Reusability (Cycles)	References
ZnMOF FeMOF	Thiamphenicol	20	SAFe@HSC carbon paper	20 mA cm^−2^	100	60	6	[72]
MIL-101(Fe)	Rhodamine B dimethyl phthalate Methylene blue Orange II	10	Fe_3_N@NG/NC	−0.2 V	97–100	60	6	[73]
MIL-100(Fe) MIL-53(Fe)	Bisphenol A	10	Fe_2_O_3_/N-C	−034 V	100	120	4	[74]
MIL-88(Fe) MIL-101(Fe) MIL-100(Fe)	Napropamide	10	CMOF@PCM	−0.14 V	90	60	3	[75]
NH2-MIL-88(Fe)	Phenol	50	FeOx/NHPC	−0.6 V	100	120	5	[76]
MIL-101(Fe)	p-nitrophenol	50	CFP@PANI@ MIL-101 cathode	5 mA cm^−2^	100	120	10	[77]
NH2-MIL-88(Fe)	Phenol	50	FeOx/ CuNxHPC	−0.6 V	100	90	10	[78]
Fe-MOF	Sulfametoxazole	20	Ce/Fe@PC-GF	20 mA	100	120	8	[79]
Cu-MOF	Bisphenol A	10	Cu/N-C	1.0 V	90	60	10	[80]
MIL-101(Fe)	sulfamethazine	10	Fe/Fe3C@PC/catalyst	25 mA	100	60	5	[81]
Fe-MOF	Fluoxetine	15	Fe_2_S/C/catalyst	50 mA	100	60	-	[76]
MIL-88(Fe) NH2-MIL-88(Fe)	Gemfibrozil	10	Nano-ZVI@C-N/catalyst	50 mA	95	60	5	[77]
MOF(Fe/Co)	Rhodamine B		MOF(2Fe/Co)/CA	−0.9 V	100	45	6	[84]
Fe-MOF	bezafibrate	15	Febpydc	100 mA	100	90	3	[85]

**Table 2:** Summary of properties of main carbon-based CDI electrodes for desalination.

Electrode	Operation Voltage (V)	*SC* (F/g)	*ESC* (mg/g)	*SA* (m^2^/g)	Ref.
AC	1.2–1.5	48–25	5.4–8.7	621–991	[41,49,50,122,123,124]
HPC	1.2	170–320	13–34	1226–2614	[42,43,125,126,127,128]
CNT	1.2–1.4	33.4	23.9	138–371	[44,129]
Graphene	1.2	43–56	6.3–6.4	898	[130,131]

**Table 3:** Comparison of CDI performance of AC at 1.2 V but different cell configurations and operation parameters.

Operation Mode	Feedwater Flow Rate (mL/min)	Contact Area (cm^2^)	Concentration (g/L)	Time (min)	Removal Percentage (%)	Ref.
Continuous	1	12.7	0.2 (5.9) {35}	50	61.0 (39.9) {12.4}	[132]
Continuous	3	243	32	25	95.42	[133]
Continuous	3	228	35	217	-	[134]
Batch	9	121	1 (5) {15}	60 (180) {540}	>99	[135]
Continuous	0.7–1.5	121	1	7 (100)	99.6 (6.7)	[136]
Continuous	0.4	60	1	-	65	[45]
Continuous	0.3	12.7	35	15	6–18	[46]

**Table 4:** Summary of properties of MOF-based CDI electrodes for desalination. Adapted from Han et al. [46].

Electrode Material	Operation Voltage (V)	[NaCl] (mM/L)	Scan Rate, (mV/s)	*SC* (F/g)	[NaCl] (mg/L)	*ESC* (mg/g)	Specific Area (m^2^/g)	Ref.
MOF-5	1.2	500	1	N.A.	500	N.A.	883	[41]
MOF-5-PC-500	1.2	500	1	12.3	500	2.17	322	
MOF-5-PC-850	1.2	500	1	33.12	500	4.05	608	
MOF-5-PC-900	1.2	500	1	108	500	4.88	1564	
MOF-5-PC-1000	1.2	500	1	105	500	9.39	991	
Zn-Co Bimetallic MOF-C	1.4	500	5	143	500	16.6	813	[50]
Zn-MOF-C (NC)	1.4	500	5	121	500	12.3	898	
Co-MOF-C (GC)	1.4	500	5	110	500	11.4	398	
Fe-MOF-PC/GO-20	1.2	1000	2	218	500 (1000)	30.3 (37.6)	713	[137]
ZIF-8-PCP-800	1.2	1000	1	129	100	5.9	606	[138]
ZIF-8-PCP-1000	1.2	1000	1	210	100	7	830	
ZIF-8-PCP-1200	1.2	1000	1	276	100 (500)	7.7 (13.9)	1188	
ZIF-8	1.4	500	1	174	500	13.1	772	[123]
HZIF-8	1.4	500	1	215	500	20.1	848	
PZS-C	1.2	500	1	237	500	13.3	N.A.	[49]
ZIF-8-C	1.2	500	1	256	500	11.3	927	
ZIF-8@PZS-C	1.2	500	1	333	500	22.2	929	
ZICarbon	1.2	1000	10	110	58.4	3.7	825	[48]
RMCarbon	1.2	1000	10	152	58.4	6.3	1367	
ZFCarbon	1.2	1000	10	226	58.4 (292)	8.1 (13.1)	2060	
Solid-ZIF Carbon (SZC)	1.2	1000	5	171	100	5.51	935	[40]
Hollow ZIF carbon (HZC)	1.2	1000	5 (1)	214 (243)	100 (500)	8.57 (15.3)	643	

## Data Availability

Data are contained within the article.
